# Challenges of proving a causal role of somatic mutations in the aging process

**DOI:** 10.1111/acel.13613

**Published:** 2022-04-18

**Authors:** Irene Franco, Gwladys Revêchon, Maria Eriksson

**Affiliations:** ^1^ Cystic Kidney Disorders Unit Division of Genetics and Cell Biology IRCCS Ospedale San Raffaele Milan Italy; ^2^ Department of Biosciences and Nutrition Center for Innovative Medicine Karolinska Institutet Huddinge Sweden

**Keywords:** accelerated aging, ageing, aging, DNA damage, DNA repair, mutagenesis, premature aging, progeria, somatic mutations

## Abstract

Aging is accompanied by the progressive accumulation of permanent changes to the genomic sequence, termed somatic mutations. Small mutations, including single‐base substitutions and insertions/deletions, are key determinants of the malignant transformations leading to cancer, but their role as initiators of other age‐related phenotypes is controversial. Here, we present recent advances in the study of somatic mutagenesis in aging tissues and posit that the current uncertainty about its causal effects in the aging process is due to technological and methodological weaknesses. We highlight classical and novel experimental systems, including premature aging syndromes, that could be used to model the increase of somatic mutation burden and understand its functional role. It is important that studies are designed to take into account the biological context and peculiarities of each tissue and that the downstream impact of somatic mutation accumulation is measured by methods able to resolve subtle cellular changes.

## 1 THE SOMATIC MUTATION THEORY OF AGING

We know the consequences of aging at the organismal level (defects of the cardiovascular system, reduction of cognitive function, osteoporosis, deregulation of immune system, graying/loss of hair, loss of skin tone, and subcutaneous fat) and at the cell and tissue level (senescence, inability to perform cell/tissue‐specific function, reduced ability to repair damage, altered proteome, and capacity of energy production). However, we have not been able to convincingly establish whether aging is the product of multiple causes that necessarily co‐occur or a cascade initiated by a primary event.

The loss of genome integrity accompanies aging. As the DNA contains the instructions for all other processes in the cell, the idea that genomic changes could initiate other molecular modifications that participate in the aging phenotypes has fascinated generations of researchers. Already in the 50s, it was proposed that aging could be initiated by a somatic mutation mechanism and that the spontaneous accumulation of somatic mutations in the genome was causative to aging (Failla, 1958; Szilard, 1959). In support of this theory, the so‐called segmental progeroid or accelerated aging syndromes, characterized by occurrence of age‐related phenotypes in kids or young adults, are prevalently caused by inherited mutations in genes involved in DNA repair and genome maintenance (Rieckher et al., 2021). More recently, the dramatic cellular response to DNA damage has been characterized and shown to induce other cellular defects contributing to aging, including senescence, epigenetic alterations, proteostatic stress, and mitochondrial dysfunction (Schumacher et al., 2021).

**FIGURE 1:**
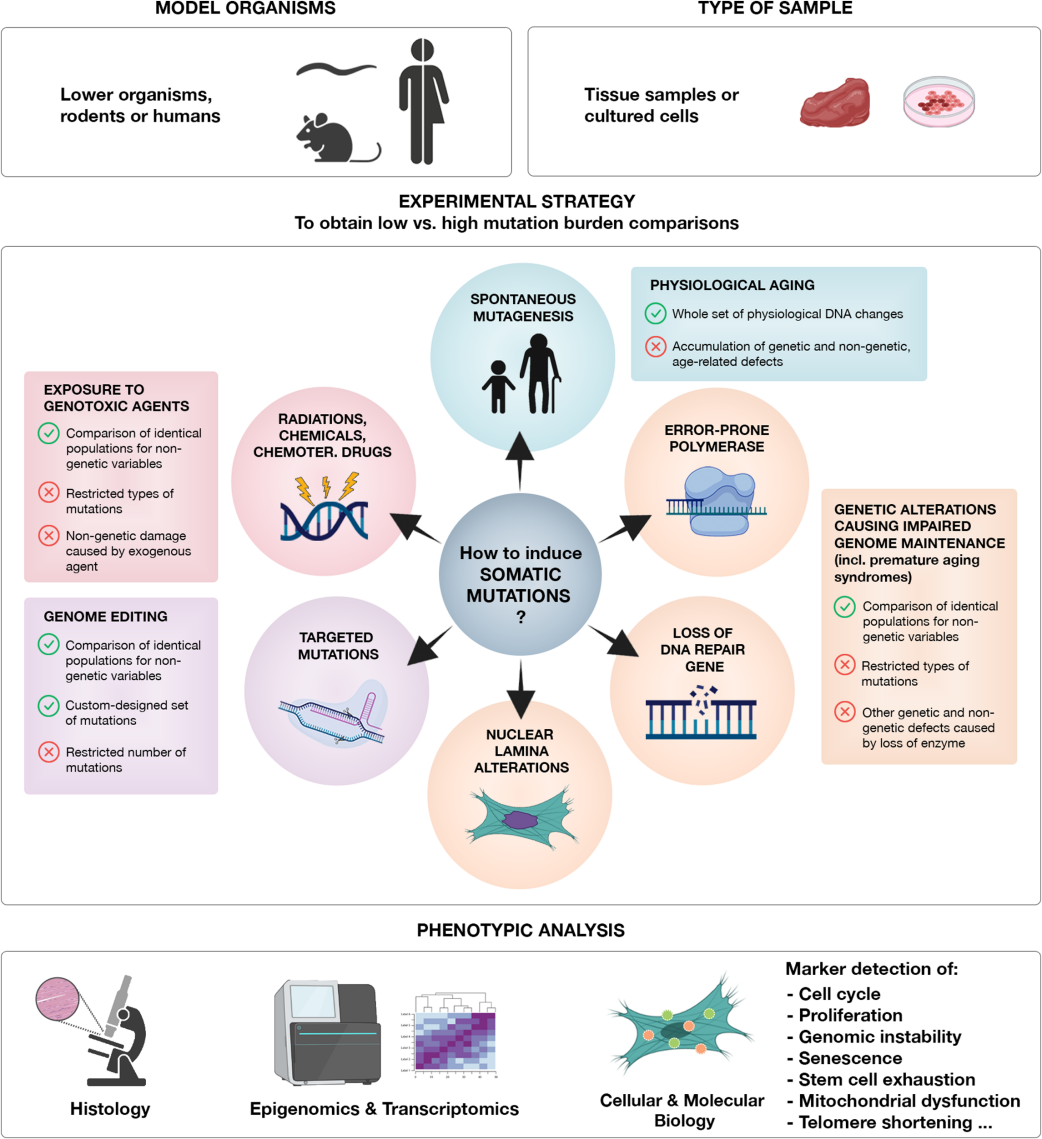
Experimental strategies and main challenges of proving a causal role of small somatic mutations in the aging process. Conclusive evidence about the role of small somatic mutations in determining aging phenotypes is still missing. The figure depicts the main experimental models, sample types, mutation strategies, and downstream analyses currently employed to address the question whether small mutations are causative to the aging process. Human syndromes naturally occurring in the population and characterized by genetic defects inducing either increased mutation burdens or accelerated aging phenotypes (premature aging syndromes) are precious models. However, to obtain answers applicable to physiological aging, the amount, types, and genomic distribution of mutations should faithfully represent the ones occurring in naturally aging tissues. In addition, the ideal experiment requires a comparison between two groups of cells/organisms that are identical, except for a lower or higher mutation burden (mimicking younger and older genomes, respectively). Such an experimental model has not been obtained yet. The main challenges are related to the establishment of the experimental groups carrying lower *vs* higher mutation burdens. Pros and cons of each experimental strategy are marked with a green thick and a red cross, respectively. A possibly promising strategy to overcome some of the limitations of currently used methods is genome editing *via* Cas12a and CRISPR arrays. This method is suitable for custom‐designed insertion of SBSs and IDs and has been implemented for simultaneous targeting of dozens to hundreds loci (Campa et al., 2019)

These arguments collectively demonstrate that a non‐physiological increase in the rate of genetic changes induces aging phenotypes. However, is the natural rate of errors occurring in physiologically aging tissues sufficient to cause appreciable phenotypes? Currently available experimental evidences do not convincingly prove that the accumulation of mutations in the genome is *per se* a cause of the phenotypes typically observed with aging. In particular, it is difficult to experimentally reproduce the genetic changes occurring during normal aging in a precise and selective way, and test their effects. First, we still do not have an exhaustive characterization of these changes. Second, inducing all the genetic changes in a well‐controlled experimental system is still unachievable. Chemical compounds or radiation introduce only a specific subset of DNA modifications. Besides, genetic changes will not be uncoupled from other cellular damages or modifications (e.g., cellular toxicity connected with oxidative damage in case of UV radiation). Inactivation of a specific gene involved in genome maintenance could cause an increase in mutations, but the loss of the associated protein function could have other cellular effects too, predictable or not. Finally, it is difficult to uncouple the effect of DNA damage (the acute lesion of the DNA) from that of DNA mutations (permanent changes in the DNA, likely initiated by DNA damage and bypassed or mis‐corrected by DNA repair). Due to the lack of such a reliable experimental setting, the characterization of the molecular events that connect the loss of genome integrity to the aging phenotypes remains challenging (Figure 1).

## 2 WHAT WE HAVE LEARNED SO FAR ABOUT SOMATIC MUTAGENESIS IN AGING TISSUES

The genome is unstable over time. It needs to be constantly duplicated, and the process may introduce errors. In addition, every cell acquires thousands of DNA lesions every day, (Lindahl, 1993) and while the repair process is efficient, it is not error‐free. The final consequence of imperfect DNA synthesis and repair is that post‐zygotic cells incorporate sequence errors, which are passed to subsequent generations. These changes are termed somatic mutations and progressively accumulate with time, eventually turning human tissues into a mosaic of slightly different genomes (Vijg, 2021; Zhang & Vijg, 2018). Multiple different types of mutations can be assessed (Zhang & Vijg, 2018). The advent of next‐generation sequencing (NGS) has revolutionized our ability to study the genome, enabling the quantitative analysis of somatic mutations. Sequencing provides information on structural variants (bigger deletions, insertions, or other rearrangements of genetic material) and shortening of chromosome extremities (telomere attrition). However, small variants, including single‐base substitutions (SBSs) and insertions/deletions of one to a few bases (IDs), are the most accurate output of the commonly used NGS technology. Thereby, our measurements of small somatic variants in normal human tissues have ramped at exceptionally high speed in the last years (Sun et al., 2021).

Major concepts established so far by the analysis of small mutations in non‐cancer genomes are listed below. All tested cells and tissues have been found to accumulate SBSs and IDs with time (Sun et al., 2021). The quantity and quality of mutations that are detected in the genome depend on the specific, combined and constant action of two processes active in the cell throughout a lifetime: DNA damage and DNA repair. Interestingly, both the cell exposure to mutagens and the lack of specific DNA repair pathways produce characteristic mutation landscapes, which have been reproduced *in vitro* (Kucab et al., 2019; Zou et al., 2021). As a consequence of the variable nature of DNA damage and repair, mutation loads vary across tissues and cell types (Blokzijl et al., 2016; Brazhnik et al., 2020; Franco et al., 2019; Li et al., 2021; Moore et al., 2021). In human tissues, an increased mutation rate has been observed upon exposure to various sources of damage, including chronic inflammation (Kakiuchi et al., 2020; Olafsson et al., 2020), bacterial infection (Lee‐Six et al., 2019; Pleguezuelos‐Manzano et al., 2020), and common lifestyle‐related mutagens, such as sunlight, alcohol, and tobacco (Saini et al., 2021; Yokoyama et al., 2019; Yoshida et al., 2020). Mutation load is not constant across the genome. Instead, there are regional differences in mutation accumulation, mainly due to the variable activity of DNA repair pathways (Supek & Lehner, 2019). As a consequence, in most cell types exons are protected from mutations compared to other regions of the genome (Blokzijl et al., 2016; Franco et al., 2018, 2019; Moore et al., 2021).

Somatic SBS and ID data from human samples are a revolutionary source of information about processes ongoing in the human body during a lifetime. For example, mutations can track early embryonic development (Bae et al., 2018; Bizzotto et al., 2021), measure the size of the stem cell population in a tissue (Lee‐Six et al., 2018) or be used to infer the mutational processes active in cells and tissues throughout life (Alexandrov et al., 2020; Supek & Lehner, 2017). For this reason, collection of somatic mutation data is of outmost importance. However, a recurrent and unanswered question persists: is the accumulation of SBSs and IDs in human cells relevant to the aging process?

Given that mitochondria are central players in the aging process, somatic mutations in mitochondrial DNA have been specifically investigated as a potential cause of age‐related phenotypes. This research has provided controversial outcomes. We refer the interested reader to recent reviews that cover the topic (Schumacher et al., 2021; Wolf, 2021), while we keep the focus of our discussion on somatic mutations in the nuclear genome. In this context, since most small variants do not impact protein structure, only a few mutations per cell may individually have significant effects on cellular processes. Nonetheless, one possibility is that SBSs and IDs collectively reduce the genome performance, acting as a “burden” that eventually impairs the cell function. Functional impairment can be due to interference with the global output of the cellular transcriptional networks, followed by a loss of cellular identity (Levy et al., 2020; Vijg & Dong, 2020). Alternatively, an excessive mutational burden may lead to induction of cell‐cycle arrest mechanisms, including senescence, aimed to prevent the transfer of unrepaired genetic information to daughter cells. This mechanism is a well‐established response to unrepaired DNA lesions (Lans et al., 2019). Permanent DNA mutations are not expected to initiate a DNA damage response. However, as discussed in above paragraphs, they are a reflection of the DNA damage/repair processes ongoing in the cell and perhaps mutations can be a sentinel of how the cell is coping with DNA damage. In support of this possibility, a change in the quality and distribution of SBSs has been observed in genomes from aged compared to young individuals. These changes are consistent with an expansion of mutations normally repaired by nucleotide excision repair (NER) and mismatch repair (MMR), and support a general decline of these DNA repair pathways with aging (Franco et al., 2019). In addition, single‐cell culture experiments of human satellite cells, the proliferative compartment of the skeletal muscle, have shown that high SBS/ID burden correlated with slower *in vitro* clonal propagation. These results may underline a causal correlation between mutation burden and dynamics of clonal expansion, mediated by higher susceptibility to senescence in cells with high mutation burden (Franco et al., 2018).

## 3 PROGEROID SYNDROMES ARE A PRECIOUS MODEL TO STUDY THE ROLE OF SOMATIC MUTATIONS IN THE AGING PROCESS

Individuals born with defects in genes involved in genome maintenance are expected to accumulate somatic mutations at a greater pace compared to the normal population. These individuals can constitute a model for testing the downstream effects of high SBS/ID burden, both at the cellular and organismal level.

Germline mutations that impair the function of proteins deputed to genome maintenance not always result in accelerated aging. In some cases, like the loss of MMR activity, the phenotype observed at organismal level is early‐onset cancer with specific tissue prevalence and a lack of evident signs of accelerated aging (Conde‐Perezprina et al., 2012). Conversely, it is true that most progeroid syndromes are caused by defects in genome maintenance. In addition to accelerated aging phenotypes, these syndromes are associated with a variable degree of cancer predisposition. Progeroid syndromes include the following: Werner, Rothmund–Thomson, and Bloom syndromes (mutations in RecQ helicases affecting DNA replication/repair/recombination, and telomere maintenance); Down syndrome (impaired DNA base excision repair [BER]); Cockayne syndrome and Trichothiodystrophy (impaired transcription‐coupled nucleotide excision repair [TC‐NER]); Xeroderma pigmentosum (global genome nucleotide excision repair [GG‐NER] defects); Ataxia telangiectasia and Fanconi anemia (impaired DNA damage response); and Hutchinson–Gilford progeria (HGPS, nuclear genome instability due to nuclear lamina dysfunction) (Niedernhofer et al., 2018; Patterson & Cabelof, 2012; Schnabel et al., 2021).

Even though progeroid syndromes have served as a precious model to study the link between the loss of genome integrity and the occurrence of aging phenotypes, the NGS revolution has only marginally interested the field. However, research on these rare diseases, especially through the use of animal models, has produced important concepts highlighting the prominent role of the genome in the aging process (Schumacher et al., 2021). An example is the loss of NER, a complex in charge of correcting DNA lesions caused by exposure to genotoxic agents such as UV light. Individuals carrying mutations that impair NER activity accumulate DNA damage in their tissues and show either cancer predisposition or premature aging phenotypes (Marteijn et al., 2014). The SBS/ID burden in these individuals is expected to be higher compared to the normal population, but actual measurements are missing. Nonetheless, reproducing this DNA repair defect in *C*. *Elegans* has evidenced that the presence of DNA lesions is a stimulus that initiates an important rearrangement of intracellular signaling pathways, metabolism, and autophagy (Edifizi et al., 2017). Many of these changes are observed in physiological aging, thus arguing that genome maintenance actively participates in determining important cellular responses and, ultimately, aging. In agreement, a general disturbance of the nuclear structure is accompanied by widespread DNA damage and produces stem cell exhaustion and accelerated aging phenotypes, including skin and bone abnormalities, fat loss, and cardiovascular aberrations, in mouse models of HGPS (Osorio et al., 2011; Sagelius et al., 2008). However, the wealth of information about phenotypic alterations in animal models of progeroid syndromes is not adequately matched by data on somatic mutagenesis, and the question whether the burden of somatic SBSs/IDs in the genome has consequences at the cellular level remains open.

## 4 ASSESSING TOLERANCE TO MUTATIONAL BURDEN

Another genetic model of enhanced mutagenesis is represented by the loss of proofreading activity of polymerases delta (POLD) and epsilon (POLE), which are in charge of DNA synthesis during cell replication. *In vitro*, proofreading activity has been shown to reduce the polymerase error rate by one order of magnitude (Schmitt et al., 2009). A recent study has quantified the number of SBSs/IDs induced by loss of proofreading activity in humans, by sequencing somatic genomes from the intestine of individuals of different ages and carrying mutated polymerases (Robinson et al., 2021). The intestinal epithelium is one of the best model systems to detect mutational burden, due to its clonal composition allowing direct assessment of mutations in the tissue. In addition, solid estimates of mutation burdens as a function of age are available for human intestinal epithelium and have been validated by different groups and technologies (Abascal et al., 2021; Blokzijl et al., 2016; Kakiuchi et al., 2020; Lans et al., 2019; Nanki et al., 2020; Pleguezuelos‐Manzano et al., 2020). Interestingly, the loss of polymerase proofreading activity can increase the mutation rates up to 7‐ and 30‐fold for SBSs and IDs, respectively (Robinson et al., 2021). The increase is so striking that teenagers with POLE mutations display the mutation burden that is expected to be found at 100 years of age in individuals with functional POLE (Robinson et al., 2021). Following the same extrapolation, 80‐year‐old individuals with POLE mutations display the mutational age of a 500‐year‐old individual.

Interestingly, individuals carrying POLD and POLE mutations do not show obvious characteristics of accelerated aging. Therefore, the authors regard these data as a proof that cells are tolerant to somatic mutations, while the decline of tissue function and aging phenotypes must be ascribed to other factors (Robinson et al., 2021). On the contrary, the study confirms that an increased mutation burden leads to cancer. In fact, these individuals experience multiple, early‐onset cancer occurrences during life, thereby the inherited disease caused by the loss of POLD and POLE proofreading activity has been termed polymerase proofreading‐associated polyposis (PPAP).

Analyses of mutation burdens in non‐cancer genomes from PPAP provide a first *in vivo* measurement of POLD and POLE proofreading activity in human tissues and its importance in protecting from cancer transformation in selected tissues. However, using the data to rule out a role of somatic mutations in determining age‐related phenotypes looks premature. Indeed, neither reliable statistics on lifespan nor a phenotypic characterization of aging phenotypes in patient tissues are available. Therefore, we actually do not know if the increased mutation burden accelerates aging at an extent that cannot be appreciated by external inspection. For example, the age‐related phenotypes of intestinal stem cells are very subtle. These defects can be appreciated only by performing specific experiments aimed to highlight, for example, reduced ability of the tissue to heal a damage, increased inflammation, or impaired capacity of single stem cells to expand and differentiate *ex vivo* (Chakravarti et al., 2021; Pentinmikko et al., 2019). The age‐related phenotype that is easily appreciated in the intestine of physiologically aging individuals, instead, is cancer. However, in agreement with cancer predisposition being a common feature of premature aging syndromes, a higher cancer incidence in PPAP can perhaps be considered a manifestation of an “accelerated aging” phenotype.

The tissue specificity of age‐related phenotypes is a key concept in the aging process. Measurements of mutation loads in PPAP patients are substantially limited to colon and endometrium (Robinson et al., 2021), two tissues undergoing subtle age‐related changes and that are also relatively spared in progeroid syndromes. A comprehensive analysis of mutation loads in the tissues that are classically studied in aging research (such as skin, fat, arteries, and brain) needs to be addressed before the PPAP model can be used to demonstrate the lack of functional effects of the SBS/ID burden. In addition, a well‐organized comparison with similar data from progeroid syndromes would constitute a great advancement for our understanding of the role of small mutations in the aging process. To date, mutation loads have been measured in the brain, muscle, and arterial wall of only one PPAP patient, showing that the excess of mutations is strongly attenuated in those tissues compared to the intestine (Robinson et al., 2021). On the contrary, sparse information is available on somatic mutation load in affected tissues from premature aging syndromes. SBS loads in individuals with accelerated aging syndromes have been measured in single neurons or in skin fibroblasts. These studies found increased mutation loads in prematurely aged tissues from patients (Lodato et al., 2018; Narisu et al., 2019). However, these data were obtained with different techniques compared to the one used in Robinson et al. (2021), thus preventing meaningful comparisons between progeroid syndromes and PPAP.

Finally, it is worth mentioning that there are examples of germline mutations in the polymerase genes that cause progeroid phenotypes in humans (Pelosini et al., 2014; Weedon et al., 2013). Different from the variants causing PPAP, these mutations leave intact or minimally affect the proofreading activity, but impair the polymerase activity of POLD1. Expected effects of the loss of POLD1‐mediated DNA synthesis include cell cycle arrest due to disturbed S phase, while the impact on somatic mutation load is unknown. Interestingly, however, individuals carrying inactive POLD1 show fat tissue atrophy, a typical feature observed in physiological aging and premature aging syndromes (Pelosini et al., 2014; Weedon et al., 2013). Comparison of somatic mutation data from different polymerase mutants would help defining the connection between disturbed DNA synthesis and age‐related phenotypes in different tissues.

## 5 TISSUE SPECIFICITY OF AGING PHENOTYPES

**FIGURE 2:**
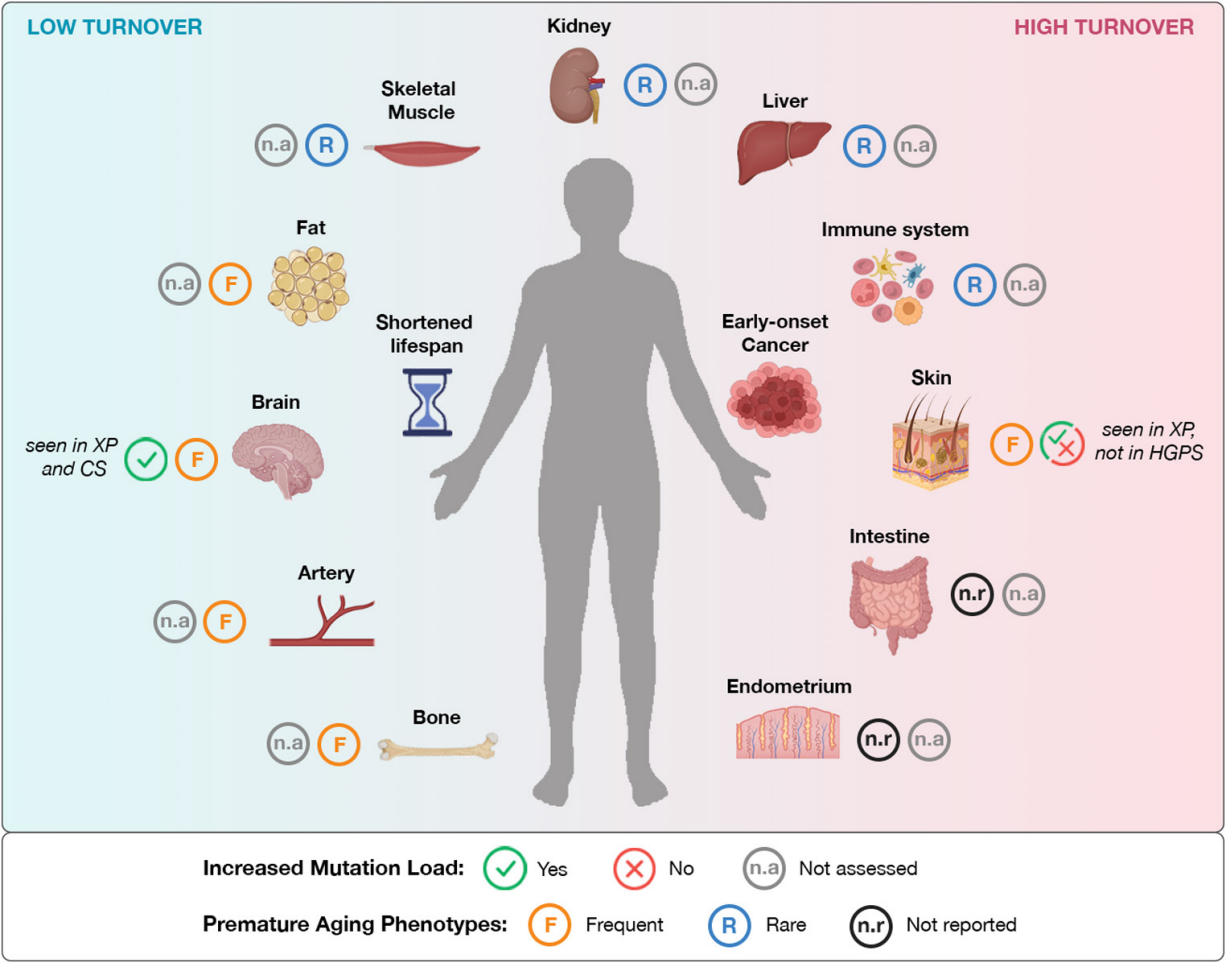
Mutation load and tissue specificity in premature aging. Most premature aging syndromes are caused by genetic defects that impair genome maintenance. However, whether these defects lead to an increased mutation load in somatic genomes of patients is mostly unexplored. Different syndromes are expected to have variable impact on somatic mutagenesis. In addition, given the segmental presentation of these disorders, somatic genomes from distinct tissues are expected to be differently affected. The figure depicts the availability of data on somatic mutation load, together with information on the likelihood (frequent, rare, and not reported) of presenting premature aging phenotypes for each organ and system. An accumulation of somatic mutations has been registered in single prefrontal cortex and hippocampal neurons derived from Xeroderma Pigmentosum (XP) and Cockayne syndrome (CS) patients (Lodato et al., 2018). Increased mutagenesis in exons of aging‐related genes was also observed in dermal fibroblasts from XP patients during *in vitro* aging, but not in Hutchinson–Gilford progeria (HGPS) fibroblasts (Narisu et al., 2019). Information on mutation load is lacking for all other tissues. Conversely, the clinical manifestations of the different premature aging syndromes are well characterized. The summary provided for every organ is obtained by manual scoring of the information reported in the NIH rare diseases database. Clinical manifestations in a specific organ were classified as “frequent” when reported in ≥40%, and “rare” when reported in <40% of premature aging syndromes taken into account (XP, CS, HGPS, Werner, Rothmund–Thomson, Bloom, Down, Trichothiodystrophy, Ataxia telangiectasia, and Fanconi anemia)

The tissue‐specific impact of the different genome maintenance pathways and the segmental nature of progeroid syndromes are crucial aspects in aging research (Figure 2). For example, the causative mutation of HGPS is a SBS that increases the activation of a cryptic splice site in the *LMNA* gene. The alternative splicing results in the expression of a truncated form of nuclear lamin A, termed progerin, which is responsible for the cellular impairments observed in HGPS individuals. Progerin compromises the nuclear structure and induces altered gene expression and persistent DNA damage. Importantly, progerin expression levels correlate with the disease severity, and increased progerin levels determine a shorter lifespan (Moulson et al., 2007). The variability is not only across individuals, but also across different tissues of the same individual. In fact, despite the mutation being present in every cell of the body, and the lamin A protein being expressed ubiquitously in differentiated cells, progerin is preferentially expressed in specific tissues (Kim et al., 2018). Tissue variability could be determined at the transcriptional level, as a consequence of cell‐type‐specific differences in the efficiency of the splice site. Alternatively, different cell types can present variable dynamics of progerin degradation (Goldman et al., 2004; Kim et al., 2018). In some tissues, such as neurons and glial cells, very low levels of progerin are detectable (Jung et al., 2012) and this associates with absence of accelerated aging phenotypes.

Other causative genes in premature aging syndromes are directly involved in the repair of DNA lesions (Lans et al., 2019; Schumacher et al., 2021). DNA exposure to damaging agents vary greatly among different tissues and cell types, and is influenced by environmental factors. Moreover, different cell types are believed to rely on different sets of DNA repair pathways that are adapted to their specific needs, as exemplified by the different importance of TC‐NER and GG‐NER in repairing DNA damage in differentiated and undifferentiated cells (Lans et al., 2010). Another key factor is tissue regeneration during aging. While some tissues, like the arterial wall, regenerate from smaller subsets of stem cells of oligoclonal or monoclonal origin, other tissues, like the skin or the colon, can rely on larger infinitive pools of stem cells. This variability is likely a key factor influencing the segmental presentation of premature aging syndromes. In the case of PPAP patients, we are assessing the effect of an enzymatic activity that is required during cell replication. Intestinal and endometrial tissues are subjected to constant renewal, supported by intense proliferation of tissue stem cells throughout life. Conversely, skeletal muscle, arteries, and brain are largely post‐mitotic and believed to undergo a very low rate of cellular turnover. Being POLE and POLD active during cell proliferation, a body wide‐lack of proofreading activity is expected to introduce a higher rate of mutations in highly proliferating cells, while the extent in post‐mitotic tissues can be multiple fold lower or even irrelevant.

## 6 CONCLUSIONS

In light of current data, we have tackled the long‐lasting question whether the burden of small mutations accumulating in the genome with aging can be responsible for the occurrence of age‐related phenotypes. We conclude that data are still missing to make any final conclusions at this time. It is, however, unlikely that somatic SBSs and IDs do not affect cellular functions. These mutations are the trigger for cancer, an age‐related disease. In this case, they are able to drive cell fate in a way that is easily recognizable, that is, clonal expansion into a macroscopic structure. It is conceivable that the burden of SBSs and IDs can also drive cell fate to more subtle states that are currently difficult to identify or detect, but are key determinants of aging phenotypes. These states include loss of differentiation, loss of plasticity, including the capacity to de‐differentiate in a context of tissue damage‐repair, loss of identity, loss of function, senescence, and apoptosis. These states may be very rare and transient in a given tissue, but able to initiate a cascade of dysfunctional mechanisms that culminate in tissue impairment. Detection of such complex and rare events is inherently difficult and might require a combination of single‐cell approaches, multi‐omics, and functional *in vivo* and *in vitro* experiments. In the context of somatic mutations, a combined assessment of somatic mutation load and other cellular parameters (transcriptional changes, activation of the DNA damage response, and cell cycle progression/arrest) is key to identify the downstream effects of genomic changes. The choice of the experimental system is also crucial, and we have described the main challenges (Figure 1). Affected tissues and cells from patients or animal models with premature aging syndromes are a valuable opportunity. Obtaining a high volume of somatic mutation data from these models of accelerated aging offers the additional opportunity to identify genes or genomic regions that are recurrently mutated or that favor clonal expansion in a tissue. These genomic loci are likely involved in the pathogenic process under investigation and could reveal potential therapeutic targets to counteract age‐related tissue remodeling. Best tools to analyze the functional relevance of these findings are generating cells and animal models carrying mutations at the identified regions and analyze these mutants under stress conditions.

How somatic mutations affect the expansion of malignant and non‐malignant clones in aging tissues is another fascinating aspect of somatic mutagenesis that is gaining increasing attention (Kakiuchi & Ogawa, 2021; Martincorena & Campbell, 2015). Tissue evolution guided by somatic mutations may constitute another universal explanation of the functional decline seen with aging. In fact, despite evident tissue differences in the appearance of aging phenotypes (Figure 2), a shared feature is a progressive increase in the occurrence of diseases, including cancer. The expansion of mutated clones in aging tissues is increasingly recognized as a factor promoting not only cancer, but also a plethora of non‐cancer diseases (Mustjoki & Young, 2021). Therefore, seventy years after the somatic mutation theory of aging was proposed, somatic genetic changes remain a plausible factor influencing the occurrence of age‐related phenotypes. A new concept that is starting to emerge is that somatic variation and the life‐long modification of each cell's genome could play a positive role in the adaptation to environmental challenges and be a critical factor shaping successful responses to tissue damage and tissue evolution. Few examples have been reported, including tissue repair during liver disease (Zhu et al., 2019), endometrial regeneration during menstrual cycle (Yamaguchi et al., 2022), and clearance of malignant clones in the esophagus (Colom et al., 2021).

In conclusion, our means to investigate the functional role of small somatic mutations have greatly improved. We are now assisting to an unprecedent step forward in our ability to detect SBSs and IDs, and have the possibility to investigate multiple tissues and multiple inherited diseases with loss of function of genes involved in genome maintenance. Measurements of somatic mutation loads are the first step. The complete picture will be obtained by exploring the biology of each tissue and the molecular implications downstream the somatic mutations. Putting together the whole spectrum of genetic, molecular, and phenotypic data will soon define the contribution of SBSs and IDs in generating aging phenotypes.

## CONFLICT OF INTEREST

The authors declare that they have no conflict of interest.

## AUTHOR CONTRIBUTION

IF wrote the manuscript with input from GR and ME; GR made the figures with input from IF and ME.

## Data Availability

The data that support the findings of this study are available from the corresponding authors upon reasonable request.
